# Supplementary figures and images for: Transcriptional Regulation of Reproductive Diapause in the Convergent Lady Beetle, Hippodamia convergens
Source: Insects. 2022 Mar 31;13(4):343. doi: 10.3390/insects13040343 (PMC9026804; doi:10.3390/insects13040343)

Early Diapause versus Mid-Diapause

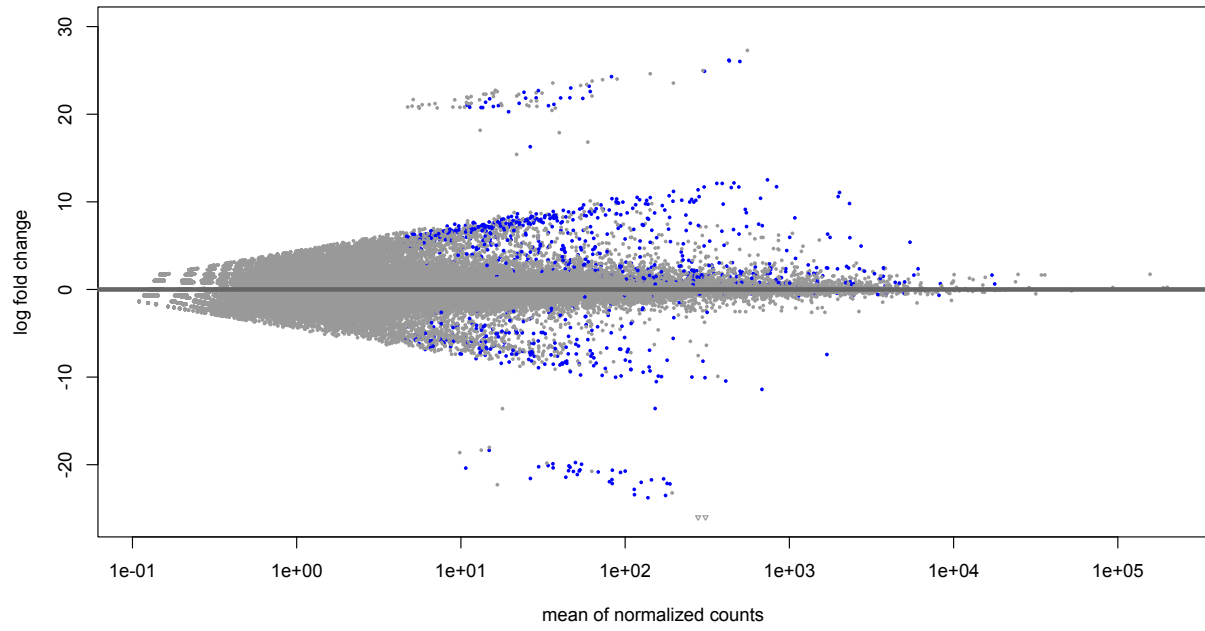

Supplement: Supplementary file 1 [file insects-13-00343-s001.zip › insects-1618331-Supplementary_Files/Supplementary_Files/Figure S1.pdf]

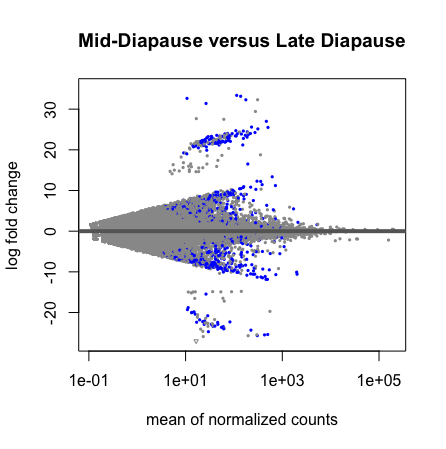

Supplement: Supplementary file 1 [file insects-13-00343-s001.zip › insects-1618331-Supplementary_Files/Supplementary_Files/Figure S2.png]
